# What could the entire cornstover contribute to the enhancement of waste activated sludge acidification? Performance assessment and microbial community analysis

**DOI:** 10.1186/s13068-016-0659-y

**Published:** 2016-11-09

**Authors:** Aijuan Zhou, Jiaguang Zhang, Kaili Wen, Zhihong Liu, Guoying Wang, Wenzong Liu, Aijie Wang, Xiuping Yue

**Affiliations:** 1 College of Environmental Science and Engineering, Taiyuan University of Technology, 030024 Taiyuan, China; 2 State Key Laboratory Breeding Base of Coal Science and Technology Co-founded by Shanxi Province and the Ministry of Science and Technology, Taiyuan University of Technology, Taiyuan, China; 3 College of Architecture and Civil Engineering, Taiyuan University of Technology, Taiyuan, China; 4 Research Center for Eco-Environmental Sciences, Chinese Academy of Sciences, Beijing, China; 5 State Key Laboratory of Urban Water Resource and Environment, Harbin Institute of Technology (SKLUWRE, HIT), Harbin, China

**Keywords:** Waste activated sludge (WAS), Corn straw (CS), Adjustment form, Volatile fatty acids (VFAs), Anaerobic digestion, Ecological estimation

## Abstract

**Background:**

Volatile fatty acids (VFAs) production from waste activated sludge (WAS) digestion is constrained by unbalanced nutrient composition (low carbon-to-nitrogen ratio). Characteristics conditioning by extra carbon sources, normally in the mixture of raw solid, has been reported to be an efficient approach to enhance WAS acidification. However, little attention has been paid to the contributions of other adjustment forms. Moreover, the corresponding ecological estimation has not been investigated yet.

**Results:**

In this study, the feasibility of corn stover (CS) conditioning with three adjustment forms [pretreated straw (S), hydrolysate (H) and hydrolysate + straw (HS)] in improving VFAs production from WAS was demonstrated. It was observed that the highest VFAs yield was achieved in H co-digesting test (574 mg COD/g VSS), while it was only 392 mg COD/g VSS for WAS digesting alone. VFAs composition was strongly adjustment form-dependent, as more acetic (HAc) and propionic (HPr) acids were generated in CS_HS and S, respectively. High-throughput sequencing analysis illustrated that acid (especially HAc)-producing characteristic genera (*Bacteroides*, *Proteiniclasticum* and *Fluviicola*) and HPr-producing characteristic genera (*Mangroviflexu*s and *Paludibacter*) were detected by CS_HS and S conditioning, respectively.

**Conclusions:**

Corn stover conditioning greatly upgraded the WAS acidification performance, especially for the CS_H adjustment form, and the VFAs yield gained was considerably larger than that previously reported. CS adjustment forms played an important role in structuring the innate microbial community in WAS. Canonical correlation analysis illustrated that characteristic genera, with better hydrolysis and acidification abilities, could be enriched by the feedstocks with certain content of cellulose, hemicellulose or their saccharification hydrolysates. Moreover, ecological estimation revealed that, as far as the entire CS (including S and H) per acre was concerned, the capacity of WAS treatment would reach that produced in a one million mts capacity wastewater treatment plants (WWTPs) per day. These findings may have crucial implications for the operation of WWTPs.

**Electronic supplementary material:**

The online version of this article (doi:10.1186/s13068-016-0659-y) contains supplementary material, which is available to authorized users.

## Background

As the by-product of wastewater treatment plants (WWTPs), 6.25 million tons of dry waste activated sludge (WAS) was produced in China in 2013, which is considered as an inevitable drawback inherent to activated sludge processes [1]. The cost of treating and disposing WAS takes 40–60% of the total costs for WWTPs [2]. With the conventional disposal routes coming under pressure, more cost-effective and environmentally benign alternatives for WAS are needed. Currently, due to its carbonaceous characteristics (organics possess 90–95% in dry weight), WAS is considered as a renewable and utilizable biomass resource and gained worldwide attention [3–5]. Cost-effective microbial conversion from WAS by anaerobic digestion to specific valuable products is an innovative and promising way to gain social and economic benefits.

As the most important intermediate, volatile fatty acids (VFAs) production from WAS digestion has been proven to be a feasible and effective carbon resource recovery process [6, 7]. Compared with time-consuming conventional energy-rich methane production (20–30 days) and the low degradation efficiency of the dry organic solids (30–50%) from WAS [8], VFAs-producing processes with proper pretreatments are completed in a relatively short operation cycle (3–8 days). Moreover, VFAs are a good choice of extra carbon for many bioprocesses, especially for biological nutrients removal (BNR) [9]. Available biodegradable carbon is required to promote the growth of denitrifying bacteria and phosphorus accumulating organisms over competing organisms. In addition, WAS contains a significant amount of embedded energy, on the order of 20 MJ/kg of dry sludge. By practicing energy recovery from WAS, the produced methane, which is clean and renewable, can be used as a substitute for fossil fuels and oil to some extent. Moreover, accumulation of VFAs was an important factor that influenced biogas generation in two-stage anaerobic digestion [10]. Regarding the way to utilize VFAs, undeniably, it mainly depends on the composition of the produced VFAs [45]. Aiming to strengthen the performance of WAS acidification, many approaches involving pretreatments (e.g., physical, chemical or biological methods) and optimizing operating conditions (e.g., pH, temperature, mixing and solids retention time) have thus been developed [8, 11, 12].

Pretreatment is a prerequisite to enhance the hydrolysis of particulate organic matter, either enclosed inside the microbial cell or enmeshed in an extracellular polymeric matrix, to soluble substrates in WAS [13]. Nevertheless, VFAs yield is still limited by the unbalanced nutrient component, especially the low carbon-to-nitrogen ratio (C/N ratio: ~6.0), caused by the large amount of proteins in WAS. This unbalanced situation results in the inefficient conversion of complex organic matter. It is concluded that the suggested C/N ratio for anaerobic sludge digestion is 20–30 [14]. The organic solid waste with a high carbon content is, therefore, suitable for being treated with WAS possessing high nitrogen content to obtain a nutrient balance. Previous research showed that conditioning with carbon-rich municipal solid wastes, agricultural residues, or industrial wastes have been reported as a cost-effective solution for the production of VFAs [15–17]. Lignocellulose is considered as an attractive raw material for the production of VFAs from co-digestion with WAS because of its availability in large quantities at low cost [18, 19]. In China, corn stover (CS) is one of the most abundant agricultural wastes [20]. CS consists of high contents of cellulose, hemicellulose and a relatively low content of lignin [21]. Recent studies have portrayed that the presence of cellulose and hemicellulose in the pretreated lignocellulose residues lead to an apparent improvement in WAS acidification, and this, in turn, may affect the composition and metabolism activity of fermentation bacteria [18]. However, a large amount of monomeric sugars (e.g., glucose, xylose and arabinose) are present in the CS hydrolysates. It is also an ideal feedstock for the carbohydrate substrate in the conversion of WAS to VFAs. It is crucial to investigate the vital roles of different CS conditioning forms on WAS acidification and the effects that will be produced on the VFAs composition and metabolism activity of fermentation bacteria. Moreover, to provide a sound basis for cost-efficient biomass stabilization and bioenergy recovery applied in WWTPs, it is important to know what the entire CS could bring for the VFAs recovery from WAS digestion.

Anaerobic digestion generally requires multiple groups of microorganisms working together to transform primary substrates to energy or high-valued chemicals [22, 23]. Previous studies showed that microbial community functional structure played an important role in bioreactor performance [22, 24, 25]. In this sense, understanding microbial behavior and interactions is essential to improve the fermentation process. Additional exploration of the microbial communities will allow engineers and researchers to establish more direct cause-and-effect relationships between community structure and function. Based on the considerations above, we investigated the VFAs production from WAS digestion by CS conditioning, with three adjustment forms (e.g., straw, hydrolysate, hydrolysate + straw), by means of process assessment associated with microbial community response analysis. We monitored the performances of hydrolysis, acidification and methanogenesis during WAS and CS co-digestion. Shifts of the acidification spectrum from WAS by conditioning with different CS forms were studied. Furthermore, we also examined the related functional microbial community structures, using high-throughput pyrosequencing of the 16S rRNA gene. Correlations between environmental variables and microbial populations were assessed, using canonical correspondence analysis (CCA).

## Methods

### Characteristics and pretreatment procedure of substrates

Waste activated sludge was collected from the Jinzhong municipal wastewater treatment plant (Taiyuan City, China) and concentrated by settling at 4 °C for 24 h. Prior to its use as feed, WAS was screened with a 1 mm sieve to remove impurities to prevent clogging problems. Ultrasonic pretreatment of WAS was performed with a 28 + 40 kHz ultrasonicator; other operation parameters were documented in a previous publication [19]. The main characteristics (average value plus standard deviation of three tests) of the concentrated and pretreated WAS are displayed in Additional file 1: Table S1.

CS used in this study was collected at Taiyuan City, Shanxi Province, China. The chopped CS was dried in the oven at 70 °C until constant weight. Then, it was milled to 2–10 mm before storing at room temperature prior to the tests. Alkaline pretreatment of CS was performed in a thermostatic water bath at 85 °C with a solid–liquid ratio of 1:10 (g dry weight to mL). The NaOH concentration was 2% (w/w) and the residence time was 1 h. The main compositions of raw and pretreated CS on a dry basis were 36 and 65% cellulose, 23 and 13% hemicellulose and 14 and 5% lignin, respectively. The CS hydrolysate contained 2.8 g/L glucose, 5.5 g/L xylose and 1.0 g/L arabinose. The volatile solids content was 0.84 g volatile solids/g dry solids. The solid residue was separated by centrifugation (10,000 rpm (9392×*g*), 10 min) (Sigma 3K30, Germany) and then dried at 70 °C to a constant weight and milled to 1–2 mm. The pretreated CS residue and supernatant were added for balancing the C/N radio of WAS fermentation.

### Experimental setup and operations

Batch experiments were carried out in twenty-one batch reactors. These reactors, with 300 mL of the mixed substrates each, were divided into seven groups (three reactors as replicates for each group). The feedstock for one group was only the ultrasonic pretreated WAS (hereinafter referred to as the control test). Two groups were fed with the mixtures of pretreated WAS and pretreated straw, with CS proportion of 50 and 35% (hereinafter referred to as the 50:50%_S and 65:35%_S tests). Two groups were fed with the mixtures of pretreated WAS and CS hydrolysate (hereinafter referred to as the 50:50%_H and 65:35%_H tests), the amounts of which were in accordance with the above straw tests. The feedstocks for the remaining two groups were the mixtures of pretreated WAS and CS, both hydrolysate and straw (hereinafter referred to as the 50:50%_HS and 65:35%_HS tests). After flushing with nitrogen gas to remove oxygen, all bottles were capped, sealed, and stirred in an air-bath shaker (100 rpm) at 35 ± 1 °C.

### DNA extraction and pyrosequencing

Before DNA extraction, sludge samples were centrifuged at 8000*g* to remove the supernatant. DNA was extracted from the sludge sediments of three replicate reactors using a Soil DNA Isolation Kit (Sangon Biotech Co., Ltd.), according to the manufacturer’s instructions, and then it was pooled together. Amplicon liberates were constructed for pyrosequencing using the bacterial fused primers 341F and 805R for the V3–V4 region of the 16S rRNA gene. To achieve the sample multiplexing during pyrosequencing, barcodes were incorporated between the adaptor and forward primer. The procedure of polymerase chain reaction (PCR) was performed in our previous study [26]. After being purified and quantified, the PCR amplicon was used for pyrosequencing on an Illumina MiSeq. The raw sequences were deposited in the NCBI Short Read Archive database with accession no. SRR3602034. The adapters, barcodes, and primers in all raw sequences were trimmed to minimize the effects of random sequencing errors. Sequences shorter than 350 bp, or containing any ambiguous base calls, were removed.

The remaining sequences were clustered into operational taxonomic units (OTUs), using the 97% identity threshold (3% dissimilarity level). Alpha diversity measurements, including Shannon and Chao1 indices were calculated for each sample. Beta diversity was calculated using the distance matrices generated using the phylogenetic-based method UniFrac [27] and then visualized using principal coordinates analysis (PCoA). Finally, the OTUs networks were visualized in Cytoscape v3.2.1 for depicting the similarities and differences between the different sludge fermentation systems [28]. CCA analyses were conducted by Canoco 4.5 to examine correlations between characteristic genera and the environmental and performance measurements, including pH and the concentrations of methane, VFAs, HAc, HPr, soluble proteins (S_pr_) and carbohydrates (S_ca_). The relative abundance of 16 characteristic bacteria was used in the CCA analysis.

### Analytical methods

Sludge samples were centrifuged at 10,000 rpm (9392×*g*) after anaerobic fermentation, filtered through a 0.45 μm cellulose nitrate membrane filter and finally stored at 4 °C, prior to analysis. The determination of TSS, VSS, SCOD, TCOD, carbohydrates, and proteins was performed as previously described [29]. A total of 500 mL Cali-5-Bond™ gas-sampling bags were used to collect the biogas produced. The total volume of gas was measured using a glass syringe. Gas composition was analyzed using a gas chromatograph (GC) (4890D, Agilent) equipped with a thermal conductivity detector (TCD). The cellulose, hemicellulose and lignin contents of CS were measured using a Fiber Analyzer (ANKOM, USA). The xylose, arabinose and glucose concentrations in CS hydrolysate were measured using high performance liquid chromatography (HPLC) (model e2695, Waters Co., Milford, MA). Another Agilent 7890 GC, equipped with a flame ionization detector (FID), was utilized to analyze the composition of the VFAs. VFAs production was calculated as the sum of the measured acetic (HAc), propionic (HPr), n-butyric (n-HBu), iso-butyric (iso-HBu), n-valeric (n-HVa) and iso-valeric (iso-HVa) acids. The COD conversion factors were 1.50 g COD/g protein (assumed as (C_4_H_6.1_O_1.2_N)x), 1.06 g COD/g carbohydrate (assumed as C_6_H_12_O_6_), 1.07 g COD/g HAc, 1.51 g COD/g HPr, 1.82 g COD/g HBu, and 2.04 g COD/g HVa.

## Results and discussion

### Effect of feedstock proportions and CS adjustment forms on WAS digestion in the processes of hydrolysis, acidification and methanogenesis

In this study, WAS provided a nitrogen source from proteins, which were the main part of the flocs and CS offered carbon sources from carbohydrates in the form of monosaccharides (H) and cellulose/hemicellulose polysaccharides (S). The time-course profiles of soluble organics are shown in Additional file 2: Fig. S1. A large amount of soluble carbohydrates and proteins were observed in CS and WAS co-digestion solution after pretreatments, which were maximized in the 50:50%_HS test, i.e., 1477 ± 22 and 12995 ± 247 mg COD/L. These soluble organic compounds were rapidly consumed during the first 96 h of operation in conjunction with VFAs production, and then they stabilized at different levels with negligible variation until the end of the run. The stabilized soluble carbohydrates in the 65:35% group, for instance, were estimated to be approximately 485, 527 and 484 mg COD/L in the H, S and HS tests, and the corresponding values for proteins were measured to be 4862, 3307 and 4198 mg COD/L approx. The accumulation of soluble organics depends on their rates of production and consumption. Production was achieved through the hydrolysis of particulate organics, which mainly aggregated in extracellular polymeric substances (EPSs) and embedded in the microbial cells for WAS [30] and polymerized in the form of cellulose and hemicellulose polysaccharides for CS. On basis of the conversion of soluble organics in the subsequent acidification and methanogenesis processes, the highest conversion of carbohydrates and proteins were achieved in 50:50%_H and HS tests, which were, respectively, 20 and 4.2 times higher than that in the control. With the decrease of CS feedstock proportion, there was a slight reduction in the consumed organics (890 and 5625 mg COD/L in the 65:35%_H and HS tests). Apparently, CS adjustment, no matter what the form was, improved the consumption of protein from WAS fermentation. The same phenomenon was also observed in other studies [15, 16].

**Fig. 1:**
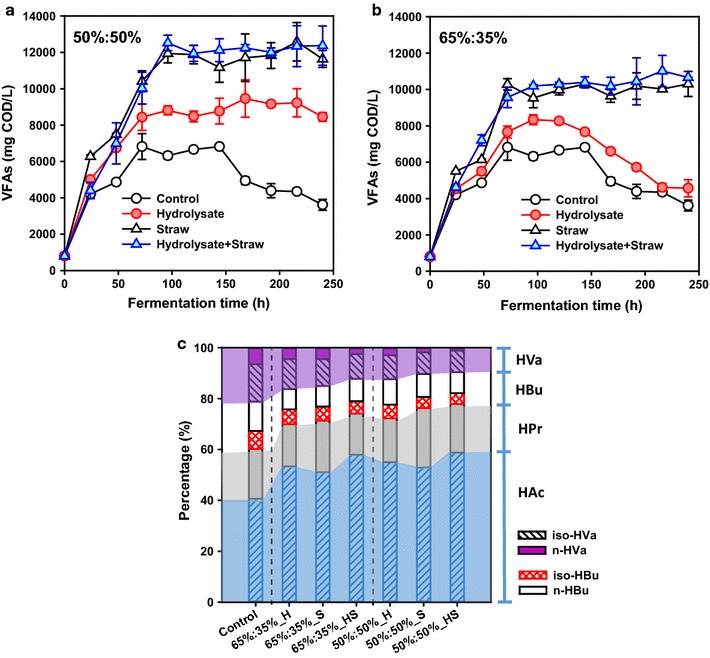
Effect of CS adjustment form on VFAs production (**a** 50:50%; **b** 65:35%) and composition (**c**) from WAS co-digestion (Note: *error bars* represent standard deviation)

VFAs were produced from proteins and carbohydrates biodegradation by acidogenic bacteria, and good agreement was obtained between them (Fig. 1 and Additional file 2: Fig. S1). From 96 h onward, VFAs production sharply increased in all tests and then fluctuated little with the further increase in fermentation time, especially for the co-digesting tests. In contrast, it gradually decreased with time extension in the control and 65: 35%_H tests. Clearly, the optimal fermentation time was 96 h for the VFAs production. This result was in good accordance with the fact that the released soluble organics were consumed at that time (Additional file 2: Fig. S1). The maximum VFAs concentration for sludge alone was only 6320 ± 196 mg COD/L. Digesting sludge alone for VFAs production has been proven to be less judicious in many cases [15, 16, 31, 32]. In comparison, in the case of co-digestion, no matter what the feedstock proportions and CS adjustment forms were, the addition of CS greatly upgraded the VFAs production. When 65: 35%_H, S and HS as carbon addition were employed, a marked rise in VFAs yields to 8338 ± 276, 9527 ± 534 and 10194 ± 72 mg COD/L were observed, respectively. A further rise in CS feedstock proportions contributed more significantly to VFAs production. The time-course curve showed that the sequence of VFAs production was HS>S>H> control tests. However, taking VFAs yield as the target, the conditioning of CS hydrolysate gained the higher value (564 mg VFAs-COD/g VSS, 50:50%), which increased 20 and 24% over that of the CS_S and HS tests. A lower CS feedstock proportion (65:35%) led to more VFAs accumulation, i.e., 583, 478 and 455 mg VFAs-COD/g VSS in the CS_H, S and HS tests, while that was only 392 mg COD/g VSS in the control.

**Fig. 2:**
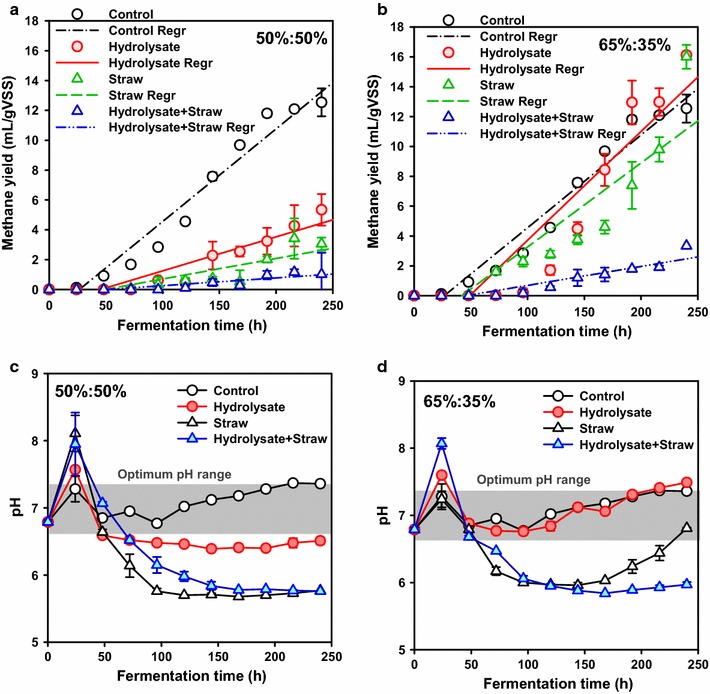
Effect of CS adjustment form on methane production from WAS (**a** 50:50%; **b** 65:35%) and the variation of pH values during WAS and CS co-digestion (**a** 50:50%; **b** 65:35%) (Note: *error bars* represent standard deviation; Panels in **c** and **d** represent the optimum pH range for methanogens)

VFAs, as the products of acidogenesis, are the substrates for methanogenesis and can be easily metabolized to methane. As depicted in Fig. 2, methane production was also strongly affected by the feedstock proportions and CS adjustment forms. We noted that, at the lower CS feedstock proportion (65:35%), (cumulative) methane production was improved by CS_H or CS_S conditioning from WAS digestion. The data obtained fitted the linear growth model (*Y*_CH4_ = constant +*kt*), and the corresponding production rate constants (*k*) were calculated. The specific methane yield slightly increased from 12.5 ± 0.9 mL/g VSS (*k* = 0.0621 h^−1^, *R* ^2^ = 0.9539) in the control to 16.0 ± 0.8 mL/g VSS (*k* = 0.0563 h^−1^, *R* ^2^ = 0.8178) and 16.1 ± 0.2 mL/g VSS (*k* = 0.0729 h^−1^, *R* ^2^ = 0.8507) in the CS_S and H tests, while it was inhibited in the CS_HS test (3.3 ± 0.0 mL/g VSS, *k* = 0.0127 h^−1^, *R* ^2^ = 0.8548) after 240 h of fermentation time. However, at the higher CS feedstock proportion (50:50%), no matter what the CS adjustment form was, methanogenesis was all indeed inhibited. Wang et al. investigated the feasibility of co-digesting sludge with *Quercus serrate* chips, and they also reported that the addition of *Quercus serrata* chips inhibited the total methane production [33]. Similarly, an adverse effect of shredded grass on methane production during the co-digestion with sludge was observed [34]. In this study, the pH reduction resulting from the high concentration of VFAs could be the reason for the low methanogenesis efficiency. It is well-known that methanogenesis is strongly pH-dependent, and most methanogenic bacteria function in a pH range of 6.5–7.2 with an optimum pH near 7.0 [35]. In this study, we have studied the pH evolution in the WAS and CS co-digestion. As shown in Fig. 2c and d, the pH values of the 50:50%_H, S and HS tests and the 65:35%_HS test at 240 h were all indeed beyond the optimum range. In contrast, the pH was in the optimum range in the 65:35%_H and control tests (7.5 and 7.4), which were consistent with the enhanced biogas yield. Interestingly, the pH value of the 65:35%_S test first decreased to ~6.0 from 96 to 168 h, which led to the slight increase in methane production. With a further increase of fermentation time, the pH value gradually increased to 6.8 (240 h), which was conducive to the metabolism of methanogens, and the corresponding methane yield sharply increased from 4.6 ± 0.4 (168 h) to 16.0 ± 0.8 mL/g VSS (240 h).

### Shift of the acidification spectrum from WAS by conditioning with different CS forms

According to the composition analysis (Fig. 1c), the six VFAs varied with different CS proportions at 96 h, when the VFAs had reached a plateau in most of the reactors. Among them, the top three (individual) VFAs produced were HAc (53–59%), HPr (17–23%) and n-HBu (8–10%) in the 50:50% tests. The results were somewhat in accordance with the observations of Jia et al. [15] and Feng et al. [16], who highlighted the positive role of perennial ryegrass and rice in sludge digestion. In contrast, the top produced VFAs were HAc (51–58%), HPr (16–20%) and iso-HVa (10–12%) in the 65:35% tests, which was consistent with individual WAS digestion (41, 20 and 15% for HAc, HPr and iso-HVa) (Fig. 1c). Similarly, this was also confirmed by our previous research [36] on the co-digestion of *Agaricus bisporus* substrates and WAS. Clearly, the product spectrum can be affected by the digesting feedstock proportions, especially for the distributions of HVa and HBu. The reason behind this maybe that HVa was mainly associated with the fermentation of proteins via reductive deamination of single amino acids or by oxidation–reduction between pairs of amino acids via the Stickland reaction [37]. The yield coefficients of HVa from monosaccharides and amino acids (i.e., ƒ_va,su_ and ƒ_va,aa_) postulated by Anaerobic Digestion Model No.1 (ADM1) were 0 and 0.23, respectively. As mentioned above, protein was the main constituent of WAS, which accounted for 58% of TCOD [19]. When digesting sludge alone, of course, the harvest was considerably more abundant in HVa (21%) than that in the conditioning tests. With the increase of the CS proportion to 35% and further to 50%, HVa was decreased to 12 and 9% in the HS groups. HBu was also abundant for digesting sludge alone (19%). With the increase of the CS proportion to 35% (50%), HBu correspondingly decreased to 14% (12%) in the HS groups. It was reported that HVa and HBu can be converted into HAc and HPr via the ß-oxidation pathway by acetogenic bacteria in the syntrophic acetogenesis process, and the specific thermodynamic equation of HBu and HVa degradation was shown as the following [37]:

1 $${\text{HBu}} + 2 {\text{H}}_{ 2} {\text{O}} \to 2 {\text{HAc}} + 2 {\text{H}}_{ 2}$$

2 $${\text{HVa}} + 2 {\text{H}}_{ 2} {\text{O}} \to {\text{HPr}} + {\text{HAc}} + 2 {\text{H}}_{ 2}$$

From an overall perspective, the conditioning of WAS digestion by external CS addition was an efficient approach for the transformation of high-molecular-weight VFAs (e.g., HVa and HBu) to low-molecular-weight VFAs (HAc and HPr).

### Overall analysis of Illumina sequence data

In total, four 16S rRNA gene libraries of the 65:35% co-digestion and control tests at 240 h were constructed from MiSeq sequencing, in total, with 101,567 high-quality reads (average length of 450 bp), and they were subsequently clustered into 11,733 OTUs at a 3% distance (Additional file 3: Table S2). Rarefaction curves for all libraries displayed shapes indicative of effective sampling of community diversity (Additional file 4: Fig. S2). The microbial diversities of the evolving communities were assessed based on α-diversity. The Shannon diversity index provided the species evenness, indicating that the control had the highest diversity (Shannon 6.29) among the four communities. Based on the Chao1 indices, in which the richness was indicated, the control also had relatively higher diversity (7663). A reduction in bacterial diversity occurred in the co-digesting tests, caused by the directional selection of bacteria, which resulted in the loss of biodiversity (Additional file 3: Table S2).

**Fig. 3:**
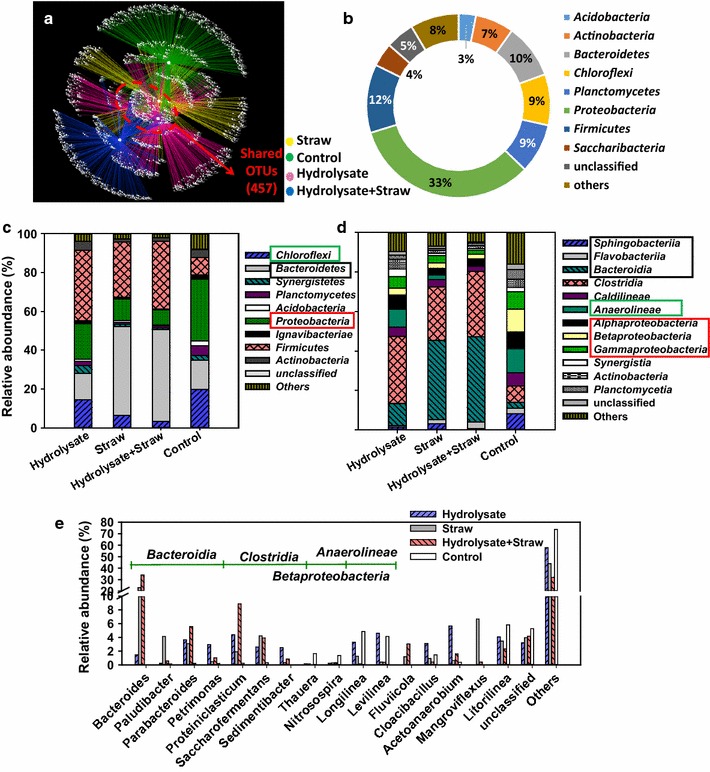
OTU networks of the bacterial communities (**a**). Overlaps of the bacterial communities based on OTU (3% distance). The shared OTUs were analyzed at phylum level (**b**). Relative abundance was defined as the number of sequences per sample. Taxonomic classification of pyrosequences from the four WAS bacterial communities at the phylum (**c**), class (**d**) and genus (**e**) levels

The similarity of the microbiome was calculated and examined by β-diversity. Differences in the bacterial community composition among the samples were assessed by PCoA, generated from unweighted UniFrac (Additional file 5: Fig. S3A). Principal components 1 and 2 explained 25.3 and 24.3% of the bacterial community composition variations, respectively. It was clear that the four samples were totally separated from each other, which was further proven by the results of hierarchical cluster analysis (HCA) (Additional file 5: Fig. S3B). That is, conditioning with CS, in different adjustment forms, substantially changed the bacterial community structure, despite the fact that the same initial source of the WAS microbial consortia was shared. To elucidate the interactions among all of the OTUs and analyze the shared and most abundant OTUs in the four WAS samples, the OTU network was constructed (Fig. 3a). Collectively, only 457 phylum-level OTUs were shared. The majority of the shared OTUs were Proteobacteria (33%), Firmicutes (12%) and Bacteroidetes (9%) (Fig. 3b).

### Microbial diversity and distribution analysis

Phylogenetic analysis of the 16S rRNA gene sequences was performed at the phylum, class and genus levels to further investigate the diversity of the microbial community. Clear changes were observed in microbial community structures during WAS co-digestion with CS in different adjustment forms (Fig. 3c, d). The phyla Firmicutes, Bacteroidetes, Chloroflexi and Proteobacteria, which were recognized as common anaerobic fermentation phyla, were dominant in the four communities. *Firmicutes* were primarily dominant in the co-digesting tests, with 36, 28 and 35% in H, S and HS, compared with 9% in the control. As reported, *Firmicutes* played a critical role in the anaerobic hydrolysis and acidification process [38]. Almost the same trend was observed for *Bacteroidetes*. Conversely, *Chloroflexi* and *Proteobacteria* decreased in all co-digesting samples. Such major difference of distributions of phyla between the sludge digestion alone and the co-digestion tests should be responsible for the distinct conditioning effects of CS addition, with different forms, on WAS digestion.

Pyrosequencing detected 67 bacterial classes in all four communities. The majority of sequences belonged to 12 classes, among which *Clostridia* (phylum *Firmicutes*) and *Bacteroidia* (phylum *Bacteroidetes*) were the dominant ones. The sum accounted for 45–76% of the total bacterial sequences for the co-digesting tests, while that was only 11% in the control (Fig. 3d). *Clostirida* was reported to consist of abundant anaerobic species and capable of decomposing solid wastes and producing organic acids by cellulolytic enzymes [39]. *Bacteroidia* was one of the few types of bacteria resistant to the extreme pH conditions, and it was reported to play a critical role in sludge reduction [40].

Further investigation on the genus level provided more detailed information about microbial communities (Fig. 3e). *Bacteroides* (belonging to the class *Bacteroidia*) took up the largest proportion of co-digesting tests, especially in S (23%) and HS (34%), and it was identified as one of the most dominant heterotrophic organisms in anaerobic wastewater treatment sludge and was capable of converting proteins and carbohydrates to HPr and HAc as its primary products in anaerobic sludge fermentation [41]. As one of class *Bacteroidia*, *Paludibacter* reached the highest abundance in S (4%), which was commonly considered to be a strictly anaerobic, HPr—producing bacterium [42]. *Mangroviflexu* (belonging to class *Marinilabiaceae*), which was also mainly detected in S (7%), was an obligately anaerobic mesophilic microorganisms and be able to ferment various substrates with the production of HPr, HAc, and succinate [43]. This was a good explanation for the higher produced HPr in CS_S conditioning test than that in H and HS (Figs. 1c, 3b). Moreover, among these class *Bacteroidia* microorganisms, *Parabacteroide* and *Petrimonas*, which were abundant in HS (6%) and H (3%), were also reported to have the ability to degrade complex organic matter to form acid [26, 44]. Three genera of *Clostridia* (*Proteiniclasticum*, *Saccharofermentans* and *Sedimentibacter*) were identified. It is worth noting that a high protein or polysaccharide content would lead to the dominance of *Proteiniclasticum* (highest in HS (9%)), which is able to produce VFAs as end products from fermentation [45]. For *Saccharofermentans*, the main end products of fermentation from glucose were HAc, lactate and fumarate [46], which peaked at ~4% in S. *Sedimentibacter*, which was mainly detected in H (3%) and only enriched at around pH 8.0, was known to ferment proteins through Stickland-type reactions to produce VFAs [47]. As stated in Fig. 2d, the pH was approximately 7.5 in the H test on day 10, which was beneficial for *Sedimentibacter* metabolism. The other dominant genera in H were *Cloacibacillus* (class *Synergistia*, 3%) and *Acetoanaerobium* (class *Bacilli*, 6%), which were specialized in amino-acid- biodegradation [48] and HAc production (from H_2_ and CO_2_) [49]. The dominant genera in the control included *Thauera* (2%), *Nitrosospira* (1%), *Longilinea* (5%) and *Levilinea* (4%), which were all common microbial consortia during the wastewater treatment.

### Correlation between environmental variables and microbial populations

**Fig. 4:**
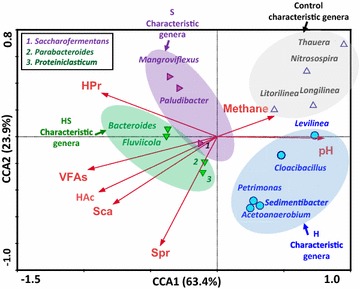
Canonical correspondence analysis (CCA) between enriched genera and environmental variables [VFAs, acetic acid (HAc), propionic acid (HPr), methane, pH, soluble proteins (S_pr_) and carbohydrates (S_ca_)]

To better understand the roles and importance of individual microbial groups in different CS and WAS co-digestion processes, plausible relationship between characteristic genera and the environmental and performance measurements, including pH, methane, VFAs, HAc, HPr, S_pr_ and S_ca_ concentrations, were evaluated using CCA (Fig. 4). Based on the assumption that the WAS digestion process is most likely driven by the predominant bacteria, we performed CCA analysis using 16 characteristic bacteria. This result can explain why different adjustment forms of CS could influence the WAS hydrolysis, acidification and methanogenesis efficiencies. The contents of methane and pH were proven to be positively correlated with the first canonical axis (explaining 63.4% of the variance of the genera distribution), and the contents of S_pr_, S_ca_, VFAs, HAc and HPr showed negative interrelations. For axis 2 (explaining 23.9% variance), only the HPr content showed good positive correlation. The detailed information is shown in Additional file 6: Table S3. The length of an arrow-line indicates the strength of the relationship between the environmental variable and the microbial community. As indicated, VFAs and HAc were strongly linked to the microbial community according to the length of the vector, followed by HPr and Sca. Moreover, the intersection angle between S_pr_ and HAc/HPr was bigger than that of S_ca_, indicating that the S_ca_ was more related to HAc and HPr production than S_pr_. This could be verified by the yield coefficients of HAc and HPr from monosaccharides and amino acids (i.e., ƒ_ac,su_, ƒ_ac,aa_, ƒ_pr,su_ and ƒ_pr,aa_), which were 0.41 vs 0.40 and 0.27 vs 0.05, respectively [37]. The changes of S_ca_ and S_pr_ concentrations were closed to VFA production (including HAc and HPr), indicating that the efficiency of WAS hydrolysis plays an important role in the subsequent acidification process. As stated, it was also found that pH was closely related to methane production, which was consistent with the above discussions (“Effect of feedstock proportions and CS adjustment forms on WAS digestion in the processes of hydrolysis, acidification and methanogenesis” section). In view of the CCA result (Fig. 4), we found that pH and methane production had very high positive correlation with some bacterial genera, including *Thauera*, *Nitrosospira*, *Longilinea*, *Litorilinea*, *Levilinea* and *Cloacibacillus*, which were abundant in the control and H tests. *Mangroviflexus* was highly correlated with HPr production, followed by *Paludibacter*, which was the characteristic genus in S. *Bacteroides* was comparatively correlated with HAc and VFAs production, followed by *Fluviicola* (the characteristic genus in HS). Instead, the characteristic genera in H were *Acetoanaerobium*, *Petrimonas* and *Sedimentibacter*, which were closely related to S_pr_. Note that these characteristic genera could be enriched by the feedstocks with certain contents of cellulose, hemicellulose (mainly in S) or their saccharification hydrolysates (mainly in H). In this sense, the CCA results suggested that adjustment forms of external carbon sources may play an important role in structuring the innate microbial community in WAS and the relationship between community structure and the measured variables may reveal the whole CS and WAS co-digestion process.

### Significances and potential implementation

This study demonstrated for the first time, the effect of CS conditioning with different adjustment forms (H, S and HS) on enhancing the WAS digestion efficiency by process assessment associated with microbial community response analysis. Experimental results showed that the highest VFAs yield was achieved by H adjustment (~574 mg COD/g VSS), followed by S (~478 mg COD/g VSS) and then by HS (~454 mg COD/g VSS), no matter what the feedstock proportion was. In contrast, it was only 392 mg COD/g VSS for the control. Clearly, the conditioning of CS greatly upgraded the WAS acidification performance. A marked rise in VFAs yield was observed when CS hydrolysate was employed. A comparison of VFAs yield from WAS digestion by co-digesting carbon-rich substrates is given in Table 1. The yield obtained in 65:35%_H was considerably larger than what has been previously reported [15, 16, 31, 32, 36, 50].

**Table 1:** Comparison of VFAs yield from WAS fermentation by co-digesting carbon-rich substrates

Sludge	Carbon-rich substrates (adjustment forms)	Feedstock proportions	VFAs yield	References
Adjusting pH (8)-treated WAS	Thermal-treated rice (TTR) (Solid)	50:50% (VSS_WAS_:VSS_TTR_)	520 mg COD/g VSS	[16]
WAS	Perennial ryegrass (Solid)	20:1 (C/N)	369 mg COD/g TS	[15]
Mixed sludge (MS)	Lime-treated bagasse (LTB) (Solid)	30:70%, 40:60% and 60:40% (g_MS_:g_LTB_)	360 mg carboxylic acid/g VS	[31]
Alkaline- thermal treated WAS	*Agaricus bisporus* substrates (ABS) (Solid)	45:55% (VSS_WAS_:VSS_ABS_)	514 mg COD/g VSS_WAS+ABS_	[36]
WAS	Sugar beet pulp lixiviation (SBPL) (Solid)	75:25% (v_SS_:v_SBPL_)	350 mg COD/g VSS	[32]
Dewatered WAS	Food waste (FW) (Solid)	12:88% (VSS_WAS_:VSS_FW_)	393 mg/g VSS	[50]
Ultrasonic treated WAS	Alkaline treated CS hydrolysate	65:35% (VSS_WAS_:VSS_CS_)	583 mg COD/g VSS	This study

VFAs composition is considered to be crucial when the WAS hydrolysate is used as an external carbon source. Among the six VFA, HAc was regarded as the favorite substrate for many bioprocesses, such as nutrient removal [51] and co-polymer production [52]. HAc was strongly dependent on adjustment form. HS conditioning groups yielded more HAc (~58%), followed by H (~54%) and S (~52%) groups. Some researchers also explored almost the same approach to produce HAc by co-fermentation with other carbon-rich substrates. Morgan-Sagastume et al. reported that the HAc ratio to other acids was higher when fermenting the mixture of primary sludge and WAS [53]. Meanwhile, HPr was also considered to be an important component for biological phosphorus removal [54]. Pijuan et al. showed that the biological removal of phosphorus was enhanced when using HPr as the sole carbon source [55]. In this study, we found that HPr was also dependent on adjustment form. That is, lower HPr was produced in the H and HS groups (~17 and ~18%), while higher HPr was produced in the S groups (22%). The sum of the concentrations of HAc and HPr was maximized in the 50:50%_HS test (78%, 9751 mg COD/L), versus 74% (7552 mg COD/L) and 60% (3803 mg COD/L) in the 65:35%_HS and control tests.

**Fig. 5:**
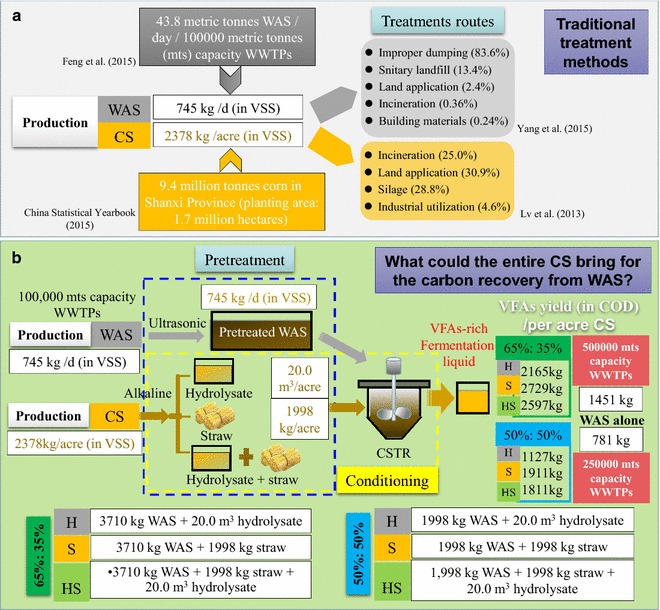
Schematic diagram of traditional treatment methods (**a**) and an enhanced concept applied in a WWTP with the WAS digestion conditioning with different CS adjustment forms (**b**)

An ecological estimation was conducted for the entire process based on the exemplary 100,000 metric tones (mts) capacity WWTPs and the corn produced in the planting area of one acre. The amount of WAS was calculated according to Feng et al. and the capacity was 43.8 mts/d (745 kg/d (in VSS)) [56]. In 2015, Shanxi Province in China generated 9.4 million tones of corn in the planting area of 1.7 million hectares [20]. Since straw is not a statistical index but rather a complex unit, we estimated the amount of straw by the ratio of residue to grain, which was reported to be approximately 1.25 for corn [57]. Then, we finally obtained the 2378 kg/acre (in VSS) capacity for CS. Currently, conventional WAS and CS disposal routes were mainly land application, incineration, landfill, silage or even improper dumping (Fig. 5a). Figure 5b demonstrates the new carbon source recovery concept, as applied in a WWTP, by WAS digestion conditioning with CS in different adjustment forms. This finding showed that it will be very considerable to harvest VFAs as a carbon source, especially for the 65:35% WAS and CS co-digesting groups. By calculation, the VFAs yield (in COD) will be 2165, 2729 and 2597 kg per acre CS with the adjustment forms of H, S and HS. More importantly, the amount of WAS treated in this case will be equivalent to that produced in a 500,000 mts capacity WWTPs per day. That will be only 1451 kg VFAs (as COD) for WAS digesting alone. Moreover, as far as the entire CS (one acre) was concerned, that is, including not only pretreated CS but also hydrolysate, the capacity of WAS treatment would achieve that is produced in a one million mts capacity WWTPs per day, where 4894 kg VFAs could be harvested. It is well-known that the removal of 1 mg nitrogen and 1 mg phosphorus consumes respectively 6–8 mg [58] and 7–10 mg COD [59]. In this case, specific removal amounts of nutrients, consuming 4894 kg VFAs, could be calculated to be at least 610 kg nitrogen or 490 kg phosphorus. Since it is expected that the problem of WAS disposal and biological treatment of nutrients will become more acute in the next decades for China, the implementation of this carbon recovery process from WAS digestion, conditioning by CS external addition, will be of increasing interest. Certainly, the practical implementation should further assess the potential challenges in WWTPs associated with management. In addition, the whole processes must be systematically developed along with life cycle, economic and ecological assessments for ensuring sustainability.

## Conclusions

CS conditioning, with three adjustment forms (S, H and HS), exerted a positive influence on VFAs production and composition during anaerobic co-digestion with WAS. A comprehensive study to shed light on the underlying mechanism was undertaken for the first time by means of process assessment associated with microbial community response analysis. CS_H conditioning gained a higher VFAs yield. Pyrosequencing revealed that the abundance of anaerobic functional microorganisms was significantly advantageous to the VFAs composition shift in three co-digesting systems. Adjustment forms of CS played an important role in structuring the innate microbial community in WAS. CCA analysis showed that the relationship between microbial community structure and the measured variables revealed the whole CS and WAS co-digestion process. Further investigation by ecological estimation revealed that the findings obtained in this study may have crucial implications for the operation of WWTPs.

## Additional files

**Additional file 1: Table S1.** The main characteristics of the substrates.

**Additional file 2: Fig. S1.** Time-course profiles of soluble proteins (A: 5%:50%; B: 65%:35%) and carbohydrates (C: 50%:50%; D: 65%:35%) during entire digestion time (Note: error bars represent standard deviation).

**Additional file 3: Table S2.** Alpha diversity of the four samples.

**Additional file 4: Fig. S2.** Rarefaction curves of bacterial communities from chemical-treated WASs based on pyrosequencing of 16S rRNA gene.

**Additional file 5: Fig. S3.** Principal coordinate analysis (PCoA) based on the unweighted UniFrac analyses (A). PC1 and PC2 axes represent 25.33% and 24.34% of the variance within the microbial community. Hierarchical cluster analysis of classified OTUs from the four WAS bacterial communities (B). The OTUs of y-axis were ordered by phylum (3% distance). Sample communities were clustered based on complete linkage method. The color intensity of scale indicates relative abundance of each OTU read.

**Additional file 6: Table S3.** The eigenvalues of first two canonical axes and their relationships with each environmental factor.

## Abbreviations

BNR: biological nutrients removal
CCA: canonical correspondence analysis
C/N: carbon-to-nitrogen
CS: corn stover
ECS: extra carbon sources
EPSs: extracellular polymeric substances
FID: flame ionization detector
GC: gas chromatography
H: hydrolysate
HAc: acetic acids
HPLC: high performance liquid chromatography
HPr: propionic acids
HS: hydrolysate + straw
iso-HBu: iso-butyric acids
iso-HVa: iso-valeric acids
mts: metric tones
n-HBu: n-butyric acids
n-HVa: n-valeric acids
OUT: operational taxonomic unit
PCR: polymerase chain reaction
S: pretreated straw
SCOD: soluble chemical oxygen demand
TCD: thermal conductivity detector
TCOD: total chemical oxygen demand
TSS: total suspended solids
VFAs: volatile fatty acids
VSS: volatile suspended solids
WAS: waste activated sludge
WWTPs: wastewater treatment plants
