# Supplementary material for: What could the entire cornstover contribute to the enhancement of waste activated sludge acidification? Performance assessment and microbial community analysis
Source: Biotechnol Biofuels. 2016 Nov 9;9:241. doi: 10.1186/s13068-016-0659-y (PMC5103463; doi:10.1186/s13068-016-0659-y)
Supplement: Supplementary file 1 — Additional file 1: Table S1. The main characteristics of the substrates. [file 13068_2016_659_MOESM1_ESM.docx]

**Table S1** The main characteristics of the substrates

| Parameter | Concentrated WAS ^a, b^ | Pretreated WAS ^a, b^ | CS hydrolysate ^a, b^ | 50%:50% co-digestion ^a, b^ | | | 65%:35% co-digestion ^a, b^ | | |
| --- | --- | --- | --- | --- | --- | --- | --- | --- | --- |
|  |  |  |  | H | S | HS | H | S | HS |
| pH | 6.68~6.90 | 6.62~6.76 | 12.21~12.46 | 10.71~10.89 | 8.98~9.07 | 10.67~10.80 | 9.39~9.51 | 7.71~7.88 | 9.65~9.79 |
| TSS (total suspended solids) | 24390±1320 | 22930±820 | - | 25660±2041 | 35190±1470 | 39070±1117 | 23040±794 | 29690±482 | 32340±2110 |
| VSS (volatile suspended solids) | 16160±980 | 15260±1140 | - | 15630±272 | 25520±440 | 27640±479 | 14290±760 | 19930±985 | 22410±1259 |
| SCOD (soluble chemical oxygen demand) | 232±27 | 4290±192 | - | 10260±791 | 6577±512 | 8343±845 | 7362±1012 | 5407±996 | 6897±443 |
| TCOD (total chemical oxygen demand) | 25420±641 | 26930±314 | 42630±227 | 30010±2991 | 38610±4790 | 42400±2799 | 28460±1455 | 31580±4198 | 35790±1177 |
| VFAs (as COD) | 69±6 | 261±33 | 888±61 | 714±141 | 1255±98 | 703±24 | 596±112 | 989±37 | 577±101 |
| Soluble carbohydrates (as COD) | 39±5 | 412±22 | 1696±407 | 1509±209 | 1366±198 | 1424±98 | 1499±112 | 1012±79 | 1207±334 |
| Soluble proteins (as COD) | 220±19 | 3130±419 | 390±21 | 13270±3419 | 7300±1112 | 10930±869 | 11525±599 | 5660±988 | 8750±1471 |

^a^ All values are expressed in mg/L except pH; ^b^ Error bars represent standard deviation.
