# Supplementary material for: What could the entire cornstover contribute to the enhancement of waste activated sludge acidification? Performance assessment and microbial community analysis
Source: Biotechnol Biofuels. 2016 Nov 9;9:241. doi: 10.1186/s13068-016-0659-y (PMC5103463; doi:10.1186/s13068-016-0659-y)
Supplement: Supplementary file 2 — Additional file 2: Fig. S1. Time-course profiles of soluble proteins (A: 5%:50%; B: 65%:35%) and carbohydrates (C: 50%:50%; D: 65%:35%) during entire digestion time (Note: error bars represent standard deviation). [file 13068_2016_659_MOESM2_ESM.docx]

**Figure S1** Time-course profiles of soluble proteins (A: 50%:50%; B: 65%:35%) and carbohydrates (C: 50%:50%; D: 65%:35%) during entire digestion time (Note: error bars represent standard deviation)
