# Supplementary material for: What could the entire cornstover contribute to the enhancement of waste activated sludge acidification? Performance assessment and microbial community analysis
Source: Biotechnol Biofuels. 2016 Nov 9;9:241. doi: 10.1186/s13068-016-0659-y (PMC5103463; doi:10.1186/s13068-016-0659-y)
Supplement: Supplementary file 3 — Additional file 3: Table S2. Alpha diversity of the four samples. [file 13068_2016_659_MOESM3_ESM.docx]

**Table S2** Alpha diversity of the four samples

| Name | Seq num*^1^ | OTU num*^2^ | Shannon index | Chao1 index | Coverage |
| --- | --- | --- | --- | --- | --- |
| Hydrolysate | 29309 | 3118 | 5.79 | 7427 | 0.9375 |
| Straw | 22495 | 2819 | 5.28 | 7055 | 0.9235 |
| Hydrolysate + Straw | 25507 | 2684 | 4.61 | 6521 | 0.9364 |
| Control | 24256 | 3112 | 6.29 | 7663 | 0.9262 |

^*1^: “Seq num” indicated the sequence numbers obtained from the high-throughput sequencing analysis;

^*2^: “OTU num” indicated the classified OTU numbers obtained from the gene sequences with the identity of over 97 %.
