# Supplementary material for: What could the entire cornstover contribute to the enhancement of waste activated sludge acidification? Performance assessment and microbial community analysis
Source: Biotechnol Biofuels. 2016 Nov 9;9:241. doi: 10.1186/s13068-016-0659-y (PMC5103463; doi:10.1186/s13068-016-0659-y)
Supplement: Supplementary file 4 — Additional file 4: Fig. S2. Rarefaction curves of bacterial communities from chemical-treated WASs based on pyrosequencing of 16S rRNA gene. [file 13068_2016_659_MOESM4_ESM.docx]

**Figure S2** Rarefaction curves of bacterial communities from chemical-treated WASs based on pyrosequencing of 16S rRNA gene.
