# Supplementary material for: What could the entire cornstover contribute to the enhancement of waste activated sludge acidification? Performance assessment and microbial community analysis
Source: Biotechnol Biofuels. 2016 Nov 9;9:241. doi: 10.1186/s13068-016-0659-y (PMC5103463; doi:10.1186/s13068-016-0659-y)
Supplement: Supplementary file 5 — Additional file 5: Fig. S3. Principal coordinate analysis (PCoA) based on the unweighted UniFrac analyses (A). PC1 and PC2 axes represent 25.33% and 24.34% of the variance within the microbial community. Hierarchical cluster analysis of classified OTUs from the four WAS bacterial communities (B). The OTUs of y-axis were ordered by phylum (3% distance). Sample communities were clustered based on complete linkage method. The color intensity of scale indicates relative abundance of each OTU read. [file 13068_2016_659_MOESM5_ESM.docx]

**Figure S3** Principal coordinate analysis (PCoA) based on the unweighted UniFrac analyses (A). PC1 and PC2 axes represent 25.33% and 24.34% of the variance within the microbial community. Hierarchical cluster analysis of classified OTUs from the four WAS bacterial communities (B). The OTUs of y-axis were ordered by phylum (3% distance). Sample communities were clustered based on complete linkage method. The color intensity of scale indicates relative abundance of each OTU read.
