# Supplementary material for: What could the entire cornstover contribute to the enhancement of waste activated sludge acidification? Performance assessment and microbial community analysis
Source: Biotechnol Biofuels. 2016 Nov 9;9:241. doi: 10.1186/s13068-016-0659-y (PMC5103463; doi:10.1186/s13068-016-0659-y)
Supplement: Supplementary file 6 — Additional file 6: Table S3. The eigenvalues of first two canonical axes and their relationships with each environmental factor. [file 13068_2016_659_MOESM6_ESM.docx]

**Table S3** The eigenvalues of first two canonical axes and their relationships with each environmental factor

|  | Axis 1 | Axis 2 |
| --- | --- | --- |
| Eigenvalues | 0.507 | 0.192 |
| Cumulative percentage variance | 63.4% | 87.3% |
| S_pr_ | -0.4258 | -0.8136 |
| S_ca_ | -0.7783 | -0.5041 |
| Methane | 0.4237 | 0.1524 |
| pH | 0.7976 | -0.0117 |
| VFAs | -0.966 | -0.2463 |
| HAc | -0.7338 | -0.6724 |
| HPr | -0.4753 | 0.7566 |
